# Modelling and analysis of the dynamics of adaptive temporal–causal network models for evolving social interactions

**DOI:** 10.1186/s40649-017-0039-1

**Published:** 2017-06-12

**Authors:** Jan Treur

**Affiliations:** Behavioural Informatics Group, Vrije Universiteit Amsterdam, Amsterdam, The Netherlands

## Abstract

**Background:**

Network-Oriented Modelling based on adaptive temporal–causal networks provides a unified approach to model and analyse dynamics and adaptivity of various processes, including mental and social interaction processes.

**Methods:**

Adaptive temporal–causal network models are based on causal relations by which the states in the network change over time, and these causal relations are adaptive in the sense that they themselves also change over time.

**Results:**

It is discussed how modelling and analysis of the dynamics of the behaviour of these adaptive network models can be performed. The approach is illustrated for adaptive network models describing social interaction.

**Conclusions:**

In particular, the homophily principle and the ‘more becomes more’ principles for social interactions are addressed. It is shown how the chosen Network-Oriented Modelling method provides a basis to model and analyse these social phenomena.

## Background

Network-Oriented Modelling has been proposed as a modelling perspective suitable for processes that are highly dynamic, circular and interactive; e.g. [1, 2]. In different application areas, this modelling perspective has been proposed in different forms: in the contexts of modelling organisations and social systems (e.g. [3–5]), of modelling metabolic processes (e.g. [6]), and of modelling electromagnetic systems (e.g. [7–9]. To address dynamics well, *Network*-*Oriented Modelling* based on *adaptive temporal*–*causal networks* has been developed [1, 2, 10]. This approach incorporates a continuous (real) time dimension. Adaptive temporal–causal network models are dynamic in two ways: their states change over time based on the causal relations in the network, but these causal relations may also change over time. As, in such networks many interrelating cycles often occur, their emerging behaviour patterns are not always easy to predict or analyse. This may make it hard to evaluate whether observed outcomes of simulations are plausible or might be due to implementation errors.

However, some specific types of properties can also be analysed by calculations in a mathematical manner, without performing simulations; see, for example [11–16]. Such properties that are found in an analytical mathematical manner can be used for *verification* of the model by checking them for the values observed in simulation experiments. If one of these properties is not fulfilled (and the mathematical analysis was done in a correct manner), then there will be some error in the implementation of the model. In this paper, methods to analyse such properties of temporal–causal network models will be described. They will be illustrated for two types for dynamic connection weights in adaptive temporal–causal network models modelling evolving social interaction: one based on the *homophily principle* (“Modelling evolving social interactions by adaptive networks based on the homophily principle” section), and one based on the *more becomes more principle* (“Modelling evolving social interactions by adaptive networks based on the ‘more becomes more’ principle” section). A preliminary, shorter presentation of part of the work described here can be found in [17].

## Network-Oriented Modelling by temporal–causal networks

The Network-Oriented Modelling approach based on temporal–causal networks, described in more detail in [1, 10] is a generic and *declarative dynamic modelling approach* based on networks of *causal relations*. Dynamics is addressed by incorporating a *continuous time dimension*. This temporal dimension enables modelling by networks that inherently contain cycles, such as networks modelling mental or brain processes, or social interaction processes, and also enables to address the timing of the processes in a differentiated manner. The modelling perspective can incorporate ingredients from different modelling approaches: for example, ingredients that are sometimes used in neural network models, and ingredients that are sometimes used in probabilistic or possibilistic modelling. It is more generic than such methods in the sense that a much wider variety of modelling elements are provided, enabling the modelling of many types of dynamical systems, as described in [1, 10]. The Network-Oriented Modelling approach is supported by a few *modelling environments* (in Matlab, or in Python, for example) that can be used to model conceptually in a declarative manner, without the need of programming. This code is in principle structure-preserving and follows the concepts described in the conceptual description presented in “Conceptual representations of temporal–causal network models” section below. It calculates simulation traces numerically based on the formulae discussed in “From a conceptual representation to a numerical representation” section, and in particular by means of the difference equations. A number of options for often-used combination functions are available within this software and can just be selected. However, for large-scale networks also, dedicated implementations can be developed directly using more efficient programming languages, or dedicated, optimised differential equation solvers developed to handle large systems of differential equations.

### Conceptual representations of temporal–causal network models

Temporal–causal network models can be represented at two levels: by a *conceptual representation* and by a *numerical representation*. A conceptual representation of a temporal–causal network model can have a *(labelled) graphical form* (or an equivalent *matrix form*), as shown in the examples presented below. The following three elements define temporal–causal networks, and are part of a conceptual representation of a temporal–causal network model:

- **connection weight ω**_***X,Y***_ Each connection from a state *X* to a state *Y* has a *connection weight* **ω**_*X,Y*_ representing the strength of the connection, often between 0 and 1, but sometimes also below 0 (negative effect).
- **combination function c**_*Y*_ **(..)** For each state *Y* (a reference to) a *combination function* **c**_*Y*_ **(..)** is chosen to aggregate the causal impacts of other states on state *Y*. This can be a standard function from a library (e.g. a scaled sum function) or an own-defined function.
- **speed factor η**_***Y***_ For each state *Y*, a *speed factor* **η**_*Y*_ is used to represent how fast a state is changing upon causal impact, usually in the [0, 1] interval.

In the first place, a conceptual representation of a temporal–causal network model involves representing in a declarative manner states and connections between them. The connections represent (causal) impacts of states on each other, as assumed to hold for the application domain addressed. Each state *X* is assumed to have an (activation) level that varies over time, indicated in the numerical representation by a real number *X(t)*. In reality, not all causal relations are equally strong, so some notion of strength of a connection from a state *X* to a state *Y* is used: a *connection weight* **ω**_*X,Y*_. Combination functions can have different forms. The applicability of a specific combination rule may depend much on the type of application addressed, and even on the type of states within an application. Therefore, for the Network-Oriented Modelling approach based on temporal–causal networks a number of standard combination functions are available as options and a number of relevant properties of such combination functions have been identified; e.g. see [10], Table 10, or [1], Chapter 2, Table 2.10. Some of these standard combination functions are scaled sum, product, complementary product, max, min, and simple and advanced logistic sum functions (for some of these examples of combination functions the numerical representations are discussed in “From a conceptual representation to a numerical representation” section). These options cover elements from different existing approaches, varying from approaches considered for reasoning with uncertainty, probability, possibility or vagueness, to approaches based on neural networks; e.g. [18–26]. In addition, there is still the option to specify any other (non-standard) combination function.

#### Conceptual representations for an adaptive network

**Fig. 1:**
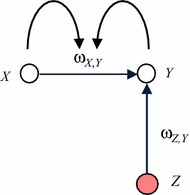
Conceptual representation of an example with an adaptive connection weight

The above three concepts (connection weight, combination function, speed factor) can be considered as parameters representing characteristics in a network model. In a non-adaptive network model, these parameters are fixed over time. But to model processes by *adaptive networks*, not only the state levels, but also these parameters can change over time. For example, the connection weights can change over time to model evolving connections in network models. For modelling processes as adaptive networks, some of the parameters (such as connection weights) are handled in a similar manner as states. For example, see Fig. 1, where the states affect the connection between them, as happens, for example, in adaptive social networks based on the homophily principle (see “Modelling evolving social interactions by adaptive networks based on the homophily principle” section).

**Fig. 2:**
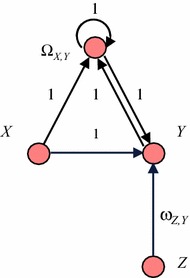
Graphical conceptual representation with state Ω_*X*,*Y*_ representing an adaptive connection weight ω_*X*,*Y*_

This can be represented differently by considering the connection weight $\upomega_{X,Y}$ as a state $\Omega_{X,Y}$ that changes over time, represented by an extra node in the network. As a first step, this node for the state $\Omega_{X,Y}$ representing $\upomega_{X,Y}$ is added and connected; see Fig. 2 for a conceptual representation. In the new situation depicted in Fig. 2, the weight $\upomega_{X,Y}$ is represented by a state $\Omega_{X,Y}$ with activation values $\Omega_{X,Y} \left( t \right)$ the same as the connection weight values ω_*X*,*Y*_(*t*) in the old situation for each *t*: $\Omega_{X,Y} \left( t \right) = \upomega_{X,Y} \left( t \right)$. This state $\Omega_{X,Y}$ is affected by both *X* and *Y*, so connections from these states to $\Omega_{X,Y}$ are incorporated. Moreover, a connection from $\Omega_{X,Y}$ to *Y* is used to represent the effect of the connection strength on *Y*, and a connection from $\Omega_{X,Y}$ to itself for persistence. The weights of all these connections are assumed 1; see Fig. 2. As a next step, it is explored what combination functions are needed for $\Omega_{X,Y}$ and *Y* in this new situation depicted in Fig. 2.

First, a combination function ${\mathbf{c}}_{{\Omega}_{X,Y}} \varvec{(..)}$ for the state $\Omega_{X,Y}$ has to be assumed, to aggregate the impacts of *X* and *Y*, and $\Omega_{X,Y}$ on $\Omega_{X,Y}$. This will depend on the adaptation principle that is chosen. Next, the new combination function for *Y* has to be determined. Below the corresponding combination functions will be discussed in more numerical detail.

### From a conceptual representation to a numerical representation

Based on a conceptual representation of a temporal–causal network model, in order to obtain a *numerical representation* of the network model the following concepts can be defined:

- $$ \text{The}\;impact\; \text{of \; state}\;X\;\text{on\;state}\;Y\;\text{at\;time}\;t\quad\quad\quad\quad\quad{\mathbf{impact}}_{X,Y} (t) $$
  The impact of state *X* on state *Y* at time *t* is defined by
  $$ {\mathbf{impact}}_{X,Y} (t) = {\boldsymbol{\upomega}}_{\varvec{X,Y}} X(t). $$
  Here *X*(*t*) is the activation level of state *X* at *t*. Note that also a connection from a state *Y* to itself is allowed. The weight $\upomega_{Y,Y}$ of such a connection can, for example, be used to model persistence of state *Y*.
- $$ \text{The}\;aggregated\; impact\; \text{on\;state}\;Y\;\text{at\;time}\;t\quad\quad\quad\quad\quad{\mathbf{aggimpact}}_{Y} (t) $$
  When more than one causal relation affects a given state *Y*, these causal effects have to be combined. To this end, some way to *aggregate multiple causal impacts* on a state is used; this is done using a *combination function* **c**_*Y*_ **(..)** that uses the impacts ${\mathbf{impact}}_{{X_{i} ,Y}} (t)$ from states *X*_1_ *, …*, *X*_*k*_ on *Y* as input and provides one *aggregated impact* value out of them:
  $$ {\mathbf{aggimpact}}_{Y} (t) = {\mathbf{c}}_{Y} ({\mathbf{impact}}_{{X_{1} ,Y}} (t), \ldots ,{\mathbf{impact}}_{{X_{k} ,Y}} (t)). $$
- Moreover, not every state has the same extent of flexibility in responding to impact; some states respond fast, and other states may be more rigid and may respond more slowly. Therefore, a *speed factor* $\boldsymbol{\upeta}_{\varvec{Y}}$ of a state *Y* is used for timing of effectuation of causal impacts, as shown in the following difference and differential equations:
  $$ \begin{aligned} Y(t +\Delta t) & = Y(t) + {\boldsymbol{\upeta}}_{Y} \left[ {{\mathbf{aggimpact}}_{Y} (t) - Y(t)} \right]\Delta t \\ {\text{d}}Y(t)/{\text{d}}t & = {\boldsymbol{\upeta}}_{Y} \left[ {{\mathbf{aggimpact}}_{Y} (t) - Y(t)} \right]. \\ \end{aligned} $$

Given the above concepts, a conceptual representation of a temporal–causal network model can be transformed in a systematic and automated manner into a numerical representation of the model, thus obtaining the following *difference* and *differential equation* for each state *Y*, expressed using the basic elements ${\boldsymbol{\upomega}}_{\varvec{X,Y}} ,{\mathbf{c}}_{\varvec{Y}} ( \ldots )$, and **η**_***Y***_ of a conceptual representation of the model:

$$ \begin{aligned} Y(t +\Delta t) & = Y(t) + {\boldsymbol{\upeta}}_{{\varvec{Y}}} \left[ {{\mathbf{c}}_{Y} \left( {{\boldsymbol{\upomega}}_{{{\varvec{X}}_{{\varvec{1}}} ,{\varvec{Y}}}} X_{ 1} \left( t \right), \ldots ,{\boldsymbol{\upomega}}_{{{\varvec{X}}_{{\varvec{k}}} ,{\varvec{Y}}}} X_{k} \left( t \right)} \right) - Y\left( t \right)} \right]\Delta t \\ {\text{d}}Y(t)/{\text{d}}t & = {\boldsymbol{\upeta}}_{{\varvec{Y}}} \left[ {{\mathbf{c}}_{{\varvec{Y}}} \left( {{\boldsymbol{\upomega}}_{{{\varvec{X}}_{{\varvec{1}}} ,{\varvec{Y}}}} X_{ 1} \left( t \right), \ldots ,{\boldsymbol{\upomega}}_{{{\varvec{X}}_{{\varvec{k}}} ,{\varvec{Y}}}} X_{k} \left( t \right)} \right) - Y\left( t \right)} \right]. \\ \end{aligned} $$

The numerical representations of some example combination functions are as follows:.

#### Numerical representation of a scaled sum combination function

In some cases, it is useful to apply a scaling factor to the sum combination function by dividing it by some *scaling factor* $\lambda$:

$$ {\mathbf{c}}(V_{ 1} ,\ldots ,V_{k} ) = {\mathbf{ssum}}_{\uplambda} (V_{ 1} ,  \ldots ,V_{k} ) = (V_{ 1} + \cdots + V_{k} )/\uplambda\text{.} $$

In cases where this combination function is used for a state *Y* with $X_{ 1} ,  \ldots , X_{k}$ connected to *Y*, then this function works as follows on the $X_{i}$:

$$ {\mathbf{ssum}}_{\uplambda} ({\boldsymbol{\upomega}}_{{\varvec{X}}_{\varvec{1}} ,{\varvec{Y}}} X_{ 1} ,  \ldots , {\boldsymbol{\upomega}}_{{\varvec{X}}_{\varvec{k}} ,{\varvec{Y}}} X_{k} ) = ({\boldsymbol{\upomega}}_{{\varvec{X}}_{\varvec{1}} ,{\varvec{Y}}} X_{ 1} + \ldots + {\boldsymbol{\upomega}}_{{\varvec{X}}_{\varvec{k}} ,{\varvec{Y}}} X_{k} )/\uplambda\text{.} $$

#### Numerical representation of a simple logistic sum combination function

The logistic sum combination function has two closely related variants, the simple variant and the more advanced variant (see below). In these functions, *τ* is a threshold parameter and *σ* a steepness parameter. The simple logistic function is defined as:

$$ {\mathbf{c}}(V_{ 1} ,  \ldots , V_{k} ) = {\mathbf{slogistic}}\;(V_{ 1} , \ldots, V_{k} ) = \frac{1}{{1 + {\mathbf{e}}\varvec{ }^{{ -\upsigma(V_{1} + \cdots + V_{\text{k}} -\uptau) }} }}. $$

To indicate the dependence of $\sigma$ and $\tau,$ sometimes these are used as subscripts: ${\mathbf{slogistic}}_{\sigma ,\tau } (V_{ 1} , \ldots ,V_{k} ).$

In cases where this combination function is used for a state *Y* with $X_{ 1} , \ldots , X_{k}$ connected to *Y*, then this function works as follows on the *X*_*i*_:

$$ {\mathbf{slogistic}}({\boldsymbol{\upomega}}_{{{\mathbf{X}}_{{\mathbf{1}}} {\mathbf{,Y}}}} X_{ 1} , \ldots , {\boldsymbol{\upomega}}_{{{\mathbf{X}}_{{\mathbf{k}}} {\mathbf{,Y}}}} X_{k} ) = 1/\left( {1 + {\text{e}}^{{ -\upsigma\left( {{\boldsymbol{\upomega}}_{{{\mathbf{X}}_{{\mathbf{1}}} {\mathbf{,Y}}}} X_{ 1} + \cdots + {\boldsymbol{\upomega}}_{{{\mathbf{X}}_{{\mathbf{k}}} {\mathbf{,Y}}}} X_{k} -\uptau} \right)}} } \right). $$

#### Numerical representation of an advanced logistic sum combination function

In the simple logistic variant, it holds **slogistic** $(0, \ldots ,0) = 1/( 1+ {\mathbf{e}}^{\sigma \tau } )$, and this is nonzero, which is undesirable property as it creates in an unintended manner activation out of no activation. This issue is compensated for in the advanced variant. The advanced logistic sum combination function is defined as

$$ {\mathbf{c}}(V_{ 1} ,  \ldots , V_{k} ) = {\mathbf{alogistic}}(V_{ 1} , \ldots , V_{k} ) = \left[ {\frac{1}{{1 + {\text{e}}^{{ - {\sigma (}V_{ 1} +  \ldots + V_{k} { - \tau )}}} }} - \frac{1}{{1 + {\text{e}}^{{ \upsigma \uptau }} }}} \right]\left( {1 + {\text{e}}^{{ -\upsigma \uptau }} } \right) $$

To indicate the dependence of $\sigma$ and $\tau,$ sometimes these are used as subscripts:

$$ {\mathbf{alogistic}}_{{\upsigma, \uptau }} (V_{ 1} , \ldots , V_{k} ) $$

For an overview of a number of standard combination functions, see Table 1.

**Table 1:** Overview of a number of standard combination functions

Name	Description	Formula **c(** *V*_1_,…, *V*_*k*_ **)**=
**sum(..)**	Sum	$V_1 + \cdots + V_k$
**product(..)** **cproduct(..)**	Product Complement product	$V_1*\cdots *V_k$ $1-(1-V_1)*\cdots *(1-V_k)$_*k*_)
**min(..)** **max(..)**	Minimal value Maximal value	**min(** *V*_1_ *,…, V*_*k*_ **)** **max(** *V*_1_ *,…, V*_*k*_ **)**
**slogistic**_*σ*,*τ*_ **(..)**	Simple logistic sum	${1 \mathord{\left/ {\vphantom {1 {\left( {1 + {\mathbf{e}}^{{ - \sigma (V_{ 1} + \cdots + V_{k} - \tau )}} } \right)}}} \right. \kern-0pt} {\left( {1 + {\mathbf{e}}^{{ - \sigma (V_{ 1} + \cdots+ V_{k} - \tau )}} } \right)}}$ with *σ*, *τ* ≥ 0
**alogistic**_*σ*,*τ*_ **(..)**	Advanced logistic sum	$\left[ {\left( {{1 \mathord{\left/ {\vphantom {1 {\left( {1 + {\mathbf{e}}^{{ - \sigma (V_{ 1} +\cdots + V_{k} - \tau )}} } \right)}}} \right. \kern-0pt} {\left( {1 + {\mathbf{e}}^{{ - \sigma (V_{ 1} + \cdots + V_{k} - \tau )}} } \right)}}} \right) - \left( {{1 \mathord{\left/ {\vphantom {1 {\left( {1 + {\mathbf{e}}^{\sigma \tau } } \right)}}} \right. \kern-0pt} {\left( {1 + {\mathbf{e}}^{\sigma \tau } } \right)}}} \right)} \right]\left( {1 + {\mathbf{e}}^{ - \sigma \tau } } \right)$ with *σ*, *τ* ≥ 0
**ssum**_*λ*_ **(..)**	Scaled sum	(*V*_1_ +⋯ + *V*_*k*_)*/λ* with *λ* > 0
**sisum(..)**	Scaled sum with interaction terms	(*V*_1_ +*⋯* + *V*_*k*_)*/λ* + Σ_*ij*_ *μ*_*ij*_ *V*_*i*_ *V*_*j*_ with *λ* > 0
**aproduct**_*β*_ **(..)**	Advanced product	*β* **cproduct(** *V*_1_ *,…, V*_*k*_ **)** + *(*1 − *β*) **product(** *V*_1_ *,…, V*_*k*_ **)** with 0 ≤ *β*≤1
**aminmax**_*β*_ **(..)**	Advanced minimum and maximum	*β* **max(** *V*_1_ *,…, V*_*k*_ **)** + (1 − *β*) **min(** *V*_1_ *,…, V*_*k*_ **)** with 0 ≤ β≤1
**aproduct-ssum**_*β*,*λ*_ **(..)**	Advanced product and scaled sum	**aproduct**_**β**_(*V*_0_, **ssum**_λ_ **(** *V*_1_ *,…, V*_*k*_ **)**) with 0 ≤ *β*≤1 and *λ* > 0

#### Numerical representations for an adaptive network

In the simple example depicted in Fig. 1, *Y* has another impact from *Z*, besides the impact from *X*. Then in the new situation depicted in Fig. 2, there are not just two but three states with impact on *Y*, namely *X, Z* and $\Omega_{X,Y}$. This requires a new combination function ${\mathbf{c}}^*_{\varvec{Y}} (V_{1} , V_{2} , W)$ for *Y* with three arguments, which is applied to the impacts $X(t),\varvec{\upomega}_{Z,X} Z(t)$ and $\Omega_{X,Y} \left( t \right)$ on *Y*, obtaining aggregated impact ${\mathbf{c}}^*_{Y} (X\left( t \right),\upomega_{Z,X} Z(t),\Omega_{X,Y} \left( t \right))$. This aggregated impact is equal to ${\mathbf{c}}_{Y} (\upomega_{X,Y} (t)X(t), {\boldsymbol{\upomega}}_{Z,Y} Z(t))$ in the previous model representation depicted in Fig. 1. Therefore,

$$ {\mathbf{c}}^*_{\varvec{Y}} (V_{ 1} ,\;V_{ 2} ,\;W) \, = \, {\mathbf{c}}_{\varvec{Y}} (WV_{ 1} ,\;V_{ 2} ) $$

For example, if in the situation of Fig. 1 ${\mathbf{c}}_{\varvec{Y}} (V_{ 1} , \, V_{ 2} )$ is the sum function $V_{ 1} + \, V_{ 2}$, then ${\mathbf{c}}^*_{\varvec{Y}} (V_{ 1} , \, V_{ 2} , \, W) = \, WV_{ 1} + \, V_{ 2}$, which is a combination of a product and a sum function. More in general, suppose in total there are *k* states *X*_*i*_ with impact on *Y*, according to combination function ${\mathbf{c}}_{\varvec{Y}} (V_{ 1} , \ldots , V_{k} )$. If all these connections are adaptive, then the new combination function becomes

$$ {\mathbf{c}}^*_{\varvec{Y}} (V_{ 1} ,\ldots , V_{k} , W_{ 1} ,\ldots , W_{k} )   =  {\mathbf{c}}_{\varvec{Y}} (W_{ 1} V_{ 1} ,\ldots ,  W_{k} V_{k} ) $$

## Modelling evolving social interactions by adaptive networks based on the homophily principle

**Fig. 3:**
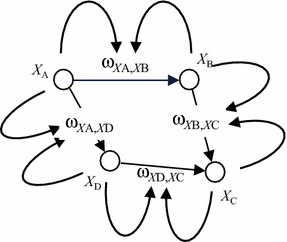
Graphical conceptual representation of an adaptive temporal–causal network model for the homophily principle

Next an adaptive temporal–causal network model is discussed to model evolving social interactions based on the homophily principle. According to this principle, also indicated as ‘birds of a feather flock together’, connections are strengthened if the connected states are similar. For example, when two persons both like the same type of music, movies, drinks, and parties, they may strengthen their connection. For the current model, the dynamic connection weights $\upomega_{{X_{A} ,X_{B} }}$ from state *X*_*A*_ of person *A* to state *X*_*B*_ of person *B* are assumed to change over time based on the principle that the closer the activation levels of the states of the interacting persons, the stronger the mutual connections between the persons will become, and the higher the difference between the activation levels, the weaker they will become. For a conceptual representation, see Fig. 3.

As discussed in “Network-Oriented Modelling by temporal–causal networks” section, $\upomega_{{X_{A} ,X_{B} }}$ can be represented by state $\Omega_{{X_{A} ,X_{B} }}$ and the weights of the connections involving $\Omega_{{X_{A} ,X_{B} }}$ are assumed 1: the weights of the connections from $X_{A}$ and $X_{B}$ to $\Omega_{{X_{A} ,X_{B} }}$, and from $\Omega_{{X_{A} ,X_{B} }}$ to $X_{B}$ and to itself. Based on this according to the temporal–causal network approach, the homophily principle may be formalised using the following general format and a combination function ${\mathbf{c}}_{A,B} (V_{ 1} , \, V_{ 2} , \, W)$ that still has to be determined:

$$ \begin{aligned} \Omega_{{X_{A} ,X_{B} }} (t + \Delta t) & = \Omega_{{X_{A} ,X_{B} }} \left( t \right) + {\boldsymbol{\upeta}}_{{\Omega_{X_{A} ,X_{B}} }} \left[ {{\mathbf{c}}_{\Omega_{{X_{A} ,X_{B} }}} (X_{A} \left( t \right),\;X_{B} \left( t \right),\;\Omega_{{X_{A} ,X_{B} }} ) - \Omega_{{X_{A} ,X_{B} }} } \right]\Delta t \\ {{{\mathbf{d}}\Omega_{{X_{A} ,X_{B} }} } \mathord{\left/ {\vphantom {{{\mathbf{d}}\Omega_{{X_{A} ,X_{B} }} } {{\mathbf{d}}t}}} \right. \kern-0pt} {{\mathbf{d}}t}} & = {\boldsymbol{\upeta}}_{\Omega_{{X_{A} ,X_{B} }}} \left[ {{\mathbf{c}}_{{\Omega_{{X_{A} ,X_{B} }} }} (X_{A} ,\;X_{B} ,\;\Omega_{{X_{A} ,X_{B} }} ) - \Omega_{{X_{A} ,X_{B} }} } \right] \\ \end{aligned} $$

Note that the connection weight $\Omega_{{X_{A} ,X_{B} }}$ increases when ${\mathbf{c}}_{{\Omega_{{X_{A}, X_{B} }} }} \left( {X_{A} \left( t \right),\;X_{B} \left( t \right),\Omega_{{X_{A} ,X_{B} }} \left( t \right)} \right)  >  \Omega_{{X_{A} ,X_{B} }} \left( t \right),$ decreases when ${\mathbf{c}}_{{\Omega_{{X_{A}, X_{B} }} }} \left( {X_{A} \left( t \right),\;X_{B} \left( t \right),\Omega_{{X_{A} ,X_{B} }} \left( t \right)} \right) < \Omega_{{X_{A} ,X_{B} }} \left( t \right)$ and stays the same when ${\mathbf{c}}_{{\Omega_{{X_{A}, X_{B} }} }} \left( {X_{A} \left( t \right),\;X_{B} \left( t \right),\Omega_{{X_{A} ,X_{B} }} \left( t \right)} \right) = \Omega_{{X_{A} ,X_{B} }} \left( t \right).$

Examples of such combination functions can be obtained when a threshold value $\tau_{{\Omega_{{X_{A} ,X_{B} }} }}$ is assumed such that the connection weight $\Omega_{{X_{A} ,X_{B} }}$ becomes stronger when $\left| {X_{A} \left( t \right) - X_{B} \left( t \right)} \right| < \tau_{{\Omega_{{X_{A} ,X_{B} }} }}$ (levels of $X_{A}$ and $X_{B}$ close to each other) and weaker when $\left| {X_{A} \left( t \right) - X_{B} \left( t \right)} \right| > \tau_{{\Omega_{{X_{A} ,X_{B} }} }}$ (levels of $X_{A}$ and $X_{B}$ not so close to each other). The following is an example which is linear in $X_{A} \left( t \right)\,{\text{and}}\,X_{B} (t)$:

$$ {\mathbf{c}}_{{\Omega_{{X_{A} ,X_{B} }} }} (X_{A} \left( t \right), \, X_{B} \left( t \right),\Omega_{{X_{A} ,X_{B} }} \left( t \right)) = \Omega_{{X_{A} ,X_{B} }} \left( t \right) \, + \beta (\tau_{{\Omega_{{X_{A} ,X_{B} }} }} - |X_{A} \left( t \right) - X_{B} \left( t \right)|) $$

The factor $\beta$ can be made dependent on $\Omega_{{X_{A} ,X_{B} }} \left( t \right)$, to keep values of $\Omega_{{X_{A} ,X_{B} }} \left( t \right)$ within the [0, 1] interval: $\beta = \alpha \Omega_{{X_{A} ,X_{B} }} \left( t \right)\left( {1 - \Omega_{{X_{A} ,X_{B} }} \left( t \right)} \right)$, with α an *amplification parameter.* This makes the combination function

$$ {\mathbf{c}}_{{\Omega_{{X_{A} ,X_{B} }} }} (V_{ 1} ,V_{ 2} , \, W) = W + \alpha W( 1- W) \, (\tau_{{\Omega_{{X_{A} ,X_{B} }} }} - \, |V_{ 1} - V_{ 2} |) $$

where $V_{ 1} ,V_{ 2}$ refer to $X_{A} ,X_{B}$ and ***W*** to $\Omega_{{X_{A} ,X_{B} }}$. Thus, we obtain the following:

$$ \begin{aligned} \Omega_{{X_{A} ,X_{B} }} (t + \Delta t) & = \Omega_{{X_{A} ,X_{B} }} (t) + {\boldsymbol{\upeta}}_{{\Omega_{{X_{A} ,X_{B} }} }} [\alpha \Omega_{{X_{A} ,X_{B} }} \left( t \right)( 1- \Omega_{{X_{A} ,X_{B} }} \left( t \right)) \, (\tau_{{\Omega_{{X_{A} ,X_{B} }} }} - |X_{A} (t) - X_{B} \left( t \right)|)]\Delta t \\ {\text{d}}\Omega_{{X_{A} ,X_{B} }} / {\text{d}}t \, & = {\boldsymbol{\upeta}}_{{\Omega_{{X_{A} ,X_{B} }} }} [\alpha \Omega_{{X_{A} ,X_{B} }} \left( t \right)( 1- \Omega_{{X_{A} ,X_{B} }} \left( t \right)) \, (\tau_{{\Omega_{{X_{A} ,X_{B} }} }} - |X_{A} (t) - X_{B} \left( t \right)|)]. \\ \end{aligned} $$

The combination function for $X_{B}$ can be found in the same way as in the “Network-Oriented Modelling by temporal–causal networks” section for *Y*.

**Fig. 4:**
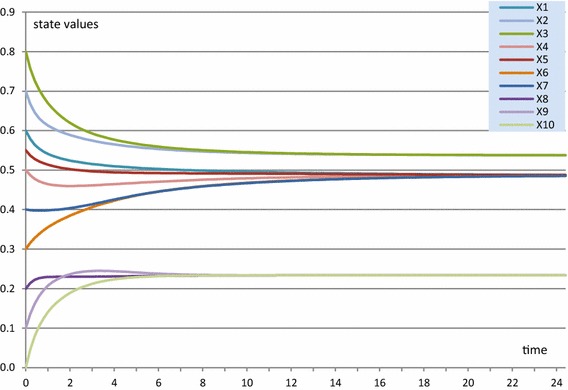
State values for the homophily example simulation showing emerging clusters

**Fig. 5:**
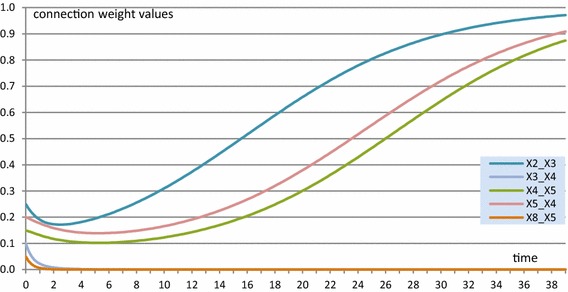
Some of the connection weights for the homophily example simulation

In Figs. 4 and 5, as an illustration, an example simulation for this homophily model is shown, based on a (fully connected) example network of 10 states *X*_1_ to *X*_10_, with the initial values of the connection weights shown in Table 2. For the contagion between states, a dynamic scaled sum function has been used in which, at each point in time, the scaling factor is equal to the sum of the connection weights involved. The homophily threshold $\tau$ was set at 0.025, and the amplification factor *α* at 20. Speed factors for states were 0.5 and for connections 0.3.

**Table 2:**
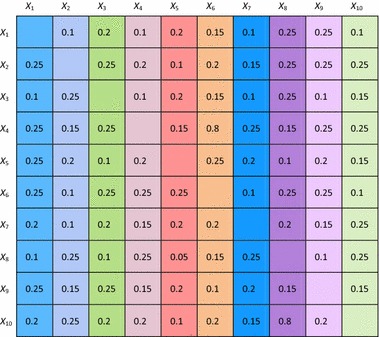
Initial connection weights for the homophily example simulation

All connection weights approximate either 0 or 1, as can be seen for a few examples (of the 90 connections) in Fig. 5. In Fig. 4, it is shown that clustering emerges, in this case in 3 clusters that in the end each are fully connected by connection weights 1, and the connection weights between states from different clusters have become practically 0. That such patterns always occur will be analysed further in the “Mathematical analysis for the homophily principle” section.

## Modelling evolving social interactions by adaptive networks based on the ‘more becomes more’ principle

**Fig. 6:**
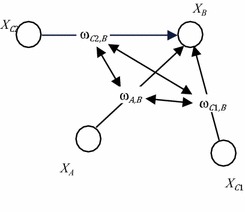
Conceptual representation of an adaptive temporal–causal network model for the ‘more becomes more’ principle

Another type of model for a dynamic connection from a person *B* to *A* takes into account to which extent other persons *C* connect to person *A*. The idea behind this is that somebody who is very popular seems worth connecting to. Sometimes this is called the ‘more becomes more’ principle, and in a wider context it relates to what sometimes is called ‘the rich get richer’ (Simon [27]), ‘cumulative advantage’ (Price [28]), ‘the Matthew effect’ (Merton [29]) or ‘preferential attachment’ (Barbasi [30]). For example, if *B* is followed by many others *C* on Twitter, then *B* seems to be interesting to follow for *A* as well. As the connections of others to *B* may change over time, this will imply that *A* also will have a dynamic connection to *B*, and in turn this connection will affect the connection of others to *B* over time as well. This can be modelled taking into account the weights $\upomega_{{C_{i} ,B}}$ for *i* = 1,…*, k* of all connections from others *C*_*i*_ to *B* as shown in Fig. 6 in conceptual representation and in numerical representation as follows:

$$ \begin{aligned} {\mathbf{d}}\upomega_{A,B} /{\mathbf{d}}t & = {\boldsymbol{\upeta}}_{\varvec{A,B}} [{\mathbf{c}}_{\varvec{A,B}} (\upomega_{{C_{1} ,B}} \ldots ,\upomega_{{C_{k} ,B}} ) - \upomega_{A,B} ] \\ \upomega_{A,B} (t + \Delta t) & = \upomega_{A,B} \left( t \right) + {\boldsymbol{\upeta}}_{\varvec{A,B}} [{\mathbf{c}}_{\varvec{A,B}} (\upomega_{{C_{1} ,B}} \left( t \right), \ldots ,\upomega_{{C_{k} ,B}} \left( t \right)) - \upomega_{A,B} \left( t \right)]. \\ \end{aligned} $$

Here **c**_***A,B***_ **(…)** is a combination function for the values $\upomega_{{C_{1} ,B}} , \ldots ,\upomega_{{C_{k} ,B}}$; for example, a logistic sum function or a scaled sum function with scale factor the number *k* of other persons connected to *B*. Note that the latter combination function only takes into account the average strengths of the connections, not the total number of them.

$$ \begin{aligned} {\mathbf{ssum}}_{\uplambda} (V_{ 1} , \ldots ,V_{k} ) & = (V_{ 1} + \cdots + V_{k} )/{\uplambda} \\ {\mathbf{alogistic}}(V_{ 1} , \ldots ,V_{k} ) & = \left[ {\frac{1}{{1 + {\mathbf{e}}\varvec{ }^{{ - \sigma (V_{1} + \cdots + V_{\text{k}} - \tau )}}  }} - \frac{1}{{1 + {\mathbf{e}}^{\sigma \tau } \varvec{ } }}} \right]( 1+ {\mathbf{e}}^{ - \sigma \tau } ). \\ \end{aligned} $$

Note that a network modelling the initiation of connections is not automatically a network indicating social contagion; this will depend on the application considered. For example, a network modelling a connection from *A* to *B* when *A is following B* on Twitter will not play a role in social contagion from *A* to *B*. For social contagion, the opposite network plays a role where a connection from *A* to *B* occurs when *A is followed by B*, which is not initiated by *A* but by *B*: on Twitter and most other social media you cannot appoint your own followers. As another example, when *A* often contacts *B* for advice, and this advice is often taken over by *A*, then the initiation connection is from *A* to *B* but the contagion connection is from *B* to *A*. In other cases, it may be different. For example, if *A* wants to announce an event or new product, he or she can choose an occasion where many others will see the message; for example, posting it on a suitable forum; in such a case both the initiation and the social contagion are directed from *A* to the others.

## Mathematical analysis of temporal–causal network models

In this section, we discuss how some types of dynamic properties of adaptive temporal–causal network models can be analysed mathematically, in particular, stationary points and monotonicity. These are basic concepts that also can be found in [1], chapter 12 or [31] . A stationary point of a state occurs at some point in time if for this time point no change occurs: the graph is horizontal at that point. Stationary points are usually maxima or minima (peaks or dips) but sometimes also other stationary points may occur. An equilibrium occurs when for all states no change occurs. From the difference or differential equations describing the dynamics for a model, it can be analysed when stationary points or equilibria occur. Moreover, it can be found when a certain state is increasing or decreasing, when a state is not in a stationary point or equilibrium. First a definition for these notions.

### Definition (stationary point, increase, decrease, and equilibrium)

- A state *Y* has a *stationary point* at *t* if ${\mathbf{d}}Y\left( t \right)/{\mathbf{d}}t = 0.$
- A state *Y* is *increasing* at *t* if ${\mathbf{d}}Y\left( t \right)/{\mathbf{d}}t > 0.$
- A state *Y* is *decreasing* at *t* if ${\mathbf{d}}Y\left( t \right)/{\mathbf{d}}t < 0.$

The model is in *equilibrium* at *t* if every state *Y* of the model has a stationary point at *t*. This equilibrium is *attracting* when for any state *Y*, all values of *Y* in some neighbourhood of the equilibrium value increase when the value is below the equilibrium value and decrease when the value is above the equilibrium value.

A question that can be addressed is whether observations based on one or more simulation experiments are in agreement with a mathematical analysis. If it is found out that the observations are in agreement with the mathematical analysis, then this provides some extent of corroboration that the implemented model is correct. If they turn out not to be in agreement with the mathematical analysis, then this indicates that probably there is something wrong, and further inspection and correction has to be initiated. Considering the differential equation for a temporal–causal network model, more specific criteria can be found:

$$ {\text{d}}Y\left( t \right)/{\text{d}}t \, = {\boldsymbol{\upeta}}_{Y} [{\mathbf{c}}_{Y} ({\boldsymbol{\upomega}}_{{X_{1} ,Y}} X_{ 1} \left( t \right), \, \ldots ,{\boldsymbol{\upomega}}_{{X_{k} ,Y}} X_{k} \left( t \right)) \, - Y\left( t \right)] $$

where *X*_1_,…*, X*_*k*_ are the states with connections to *Y*. For example, it can be concluded that

$$ {\text{d}}Y\left( t \right)/{\text{d}}t > 0 \Leftrightarrow {\mathbf{c}}_{Y} ({\boldsymbol{\upomega}}_{{X_{1} ,Y}} X_{ 1} \left( t \right), \, \ldots ,{\boldsymbol{\upomega}}_{{X_{k} ,Y}} X_{k} \left( t \right)) > Y\left( t \right). $$

In this manner, the following criteria can be found:

### Criteria for increase, decrease, stationary point and equilibrium

Let *Y* be a state and $X_{ 1} , \ldots ,X_{k}$ the states connected toward *Y*. Then, the following hold:

$$\begin{aligned}Y\;\text{has\;a\;stationary\;point\;at}\;t & \Leftrightarrow{\mathbf{c}}_{Y} ({\boldsymbol{\upomega}}_{{X_{1} ,Y}} X_{ 1} \left( t \right), \, \ldots ,{\boldsymbol{\upomega}}_{{X_{k} ,Y}} X_{k} \left( t \right)) = Y(t) \\Y\;\text{is\;increasing\; at}\;t& \Leftrightarrow{\mathbf{c}}_{Y} ({\boldsymbol{\upomega}}_{{X_{1} ,Y}} X_{ 1} \left( t \right), \, \ldots ,{\boldsymbol{\upomega}}_{{X_{k} ,Y}} X_{k} \left( t \right)) > Y(t) \\Y\;\text{is\;decreasing\; at}\;t& \Leftrightarrow{\mathbf{c}}_{Y} ({\boldsymbol{\upomega}}_{{X_{1} ,Y}} X_{ 1} \left( t \right), \, \ldots ,{\boldsymbol{\upomega}}_{{X_{k} ,Y}} X_{k} \left( t \right)) < Y(t)\\\text{The\;model\;is\;in\;equilibrium\;a}\;t& \Leftrightarrow {\mathbf{c}}_{Y} ({\boldsymbol{\upomega}}_{{X_{1} ,Y}} X_{ 1} \left( t \right), \, \ldots ,{\boldsymbol{\upomega}}_{{X_{k} ,Y}} X_{k} \left( t \right)) = Y(t)\; \text{for}\;\boldsymbol{\it{every}}\;\text{state}\;Y(\text{i.e.\;a\;joint\;stationary\;state})\\\end{aligned}$$

Note that these criteria can immediately be found from a conceptual representation of a temporal–causal network model, as long as the referred combination function is known. Using the above criteria, no further numerical representation is needed of the difference or differential equations, for example. From these criteria, more insight can be obtained about the behaviour of the network model, in particular which stationary points are possible for a state in the model, and which equilibria are possible for the whole model. Sometimes, the stationary point equation can be rewritten into an equation of the form *Y(t)* = .. such that *Y(t)* does not occur on the right-hand side. In the “Mathematical analysis for the homophily principle” and “Mathematical analysis for the ‘more becomes more’ principle” sections, examples of this are shown.

The criteria can also be used to verify (the implementation of) the model based on inspection of stationary points or equilibria, in two different manners A and B. Note that in a given simulation the stationary points that are identified are usually approximately stationary; how closely they are approximated depends on different aspects, for example, on the step size, or on how long the simulation is done.

#### A. Verification by checking stationary points through substitution of the values from a simulation in the criterion

1. Generate a simulation.
2. Consider any state *Y* with a stationary point at any time point *t* and states *X*_1_ **, …,** *X*_*k*_ affecting it.
3. Substitute the values $Y\left( t \right)$ and $X_{ 1} \left( t \right)$ **, …,** $X_{k} \left( t \right)$ in the criterion ${\mathbf{c}}_{Y} (\upomega_{{X_{1} ,Y}} X_{ 1} (t), \ldots ,\upomega_{{X_{k} ,Y}} X_{k} (t)) = Y(t).$
4. If the equation holds (for example, with an accuracy <0.05), then this test succeeds, otherwise it fails
5. If this test fails, then it has to be explored were the error can be found

Note that this method A. works without having to solve the equations, only substitution takes place; therefore it works for any choice of combination function. Moreover, note that the method also works when the values of the states fluctuate, for example according to a recurring pattern (a limit cycle). In such cases for each state, there are maxima (peaks) and minima (dips), which also are stationary points to which the method can be applied; here it is important to choose a small step size as each stationary point occurs at one time point only. There is still another method B. possible that can be applied sometimes; it is based on solving the equations for the stationary point values by symbolic rewriting. This can provide explicit expressions for stationary point values in terms of the parameters of the model. Such expressions can be used to predict equilibrium values for specific simulations, based on the choice of parameter values.

#### B. Verification by solving the equilibrium equations and comparing predicted equilibrium values to equilibrium values in a simulation

1. Consider the equilibrium equations for all states *Y*:
  $$ {\mathbf{c}}_{Y} (\upomega_{{X_{1} ,Y}} X_{ 1} (t), \ldots ,\upomega_{{X_{k} ,Y}} X_{k} (t)) = Y(t). $$
2. Leave the *t* out and denote the values as constants
  $$ {\mathbf{c}}_{Y} (\upomega_{{X_{1} ,Y}} \underline{\mathbf{X}}_{ 1} , \ldots ,\upomega_{{{X}_{k} ,Y}} \underline{\mathbf{X}}_{ k} ) = \underline{Y} . $$

An equilibrium is a solution $\underline{\mathbf{X}}_{ 1} , \ldots ,\underline{\mathbf{X}}_{ k}$ of the following set of *n* equilibrium equations in the *n* states $X_{ 1} , \, \ldots ,X_{n}$ of the model:

$$ \begin{aligned} & {\mathbf{c}}_{{X_{1} }} (\upomega_{{X_{1}, X_{1} }} \underline{{{\mathbf{X}}_{1} }} , \ldots ,\upomega_{{X_{n} ,X_{1} }} \underline{{{\mathbf{X}}_{n} }} ) = \underline{{{\mathbf{X}}_{1} }} \hfill \\ & \ldots \hfill \\ & {\mathbf{c}}_{{X_{n} }} (\upomega_{{X_{ 1} ,X_{n} }} \underline{{{\mathbf{X}}_{1} }} , \ldots ,\upomega_{{X_{n} ,X_{n} }} \underline{{{\mathbf{X}}_{n} }} ) = \underline{{{\mathbf{X}}_{n} }} \hfill \\ \end{aligned} $$

3. Solve these equations mathematically in an explicit analytical form: for each state *X*_*i*_ a mathematical formula **X**_***i***_ = … in terms of the parameters of the model (connection weights and parameters in the combination function ${\mathbf{c}}_{{X}_{i}}$ **(..)**, such as the steepness *σ* and threshold *τ* in a logistic sum combination function); more than one solution is possible.
4. Generate a simulation.
5. Identify equilibrium values in this simulation.
6. If for all states *Y*, the predicted value **Y** from a solution of the equilibrium equations equals the value for *Y* obtained from the simulation (for example, with an accuracy <0.05), then this test succeeds, otherwise it fails.
7. If this test fails, then it has to be explored where the error can be found.

For more details, see [1], chapter 12, or [31]. This method B. provides more, but a major drawback is that it cannot be applied in all situations; this depends on the chosen combination functions: e.g. for logistic functions, it does not work.

## Mathematical analysis for the homophily principle

In the “Modelling evolving social interactions by adaptive networks based on the ‘more becomes more’ principle” section, it was shown how the homophily principle for evolving social interaction may be modelled using a combination function:

$$ {\mathbf{c}}_{{\Omega_{{X_{A} ,X_{B} }} }} (V_{ 1} ,V_{ 2} ,W) = W + W\left( { 1- W} \right) \, \left( {\tau_{{\Omega_{{X_{A} ,X_{B} }} }} - |V_{ 1} - V_{ 2} |} \right) $$

In this section, we analyse which stationary points can occur for $\Omega_{{X_{A} ,X_{B} }}$, according to the approach described in “Mathematical analysis of temporal–causal network models”. For this case, the criterion from the “Mathematical analysis of temporal–causal network models” section for a stationary point is

$$ \begin{aligned} & {\mathbf{c}}_{{\Omega_{{X_{A} ,X_{B} }} }} \left( {X_{A} \left( t \right), \, X_{B} \left( t \right),\Omega_{{X_{A} ,X_{B} }} \left( t \right)} \right) = \Omega_{{X_{A} ,X_{B} }} \left( t \right) \Leftrightarrow  \hfill \\ & \Omega_{{X_{A} ,X_{B} }} \left( t \right)\left( { 1- \Omega_{{X_{A} ,X_{B} }} \left( t \right)} \right)\left( {\tau_{{\Omega_{{X_{A} ,X_{B} }} }} - |X_{A} \left( t \right) - X_{B} \left( t \right)|} \right) = 0 \hfill \\ \end{aligned} $$

Clearly, for $\Omega_{{X_{A} ,X_{B} }} (t) = 0$ or $\Omega_{{X_{A} ,X_{B} }} (t) = 1,$ one of the left-hand side factors in this condition is 0. In contrast, when $0 < \Omega_{{X_{A} ,X_{B} }} (t) < 1,$ the right-hand factor should equal 0:

$$ \tau_{{\Omega_{{X_{A} ,X_{B} }} }} - |X_{A} \left( t \right) - X_{B} \left( t \right)| = 0 \Leftrightarrow \left| {X_{A} \left( t \right) - X_{B} \left( t \right) \, } \right| = \tau_{{\Omega_{{X_{A} ,X_{B} }} }} . $$

Therefore, in principle, there are three types of stationary points for $\Omega_{{X_{A} ,X_{B} }} (t)$.

*Stationary points for* $\Omega_{{X_{A} ,X_{B} }} (t)$:

$$ \Omega_{{X_{A} ,X_{B} }} (t) = 0\,{\text{or}}\,\Omega_{{X_{A} ,X_{B} }} (t) = 1\,{\text{or}}\,|X_{A} \left( t \right) - X_{B} \left( t \right)| = \tau_{{\Omega_{{X_{A} ,X_{B} }} }} \,{\text{and}}\,\Omega_{{X_{A} ,X_{B} }} (t) \text{\,have\,any\,value}.$$

Similarly, the following can be found.

*Increasing* $$ \Omega_{{X_{A} ,X_{B} }} (t) $$

$$ {\text{d}}\Omega_{{X_{A} ,X_{B} }} (t)/{\text{d}}t > 0 \Leftrightarrow (\tau_{{\Omega_{{X_{A} ,X_{B} }} }} - |X_{A} \left( t \right) - X_{B} \left( t \right)|) > 0 \Leftrightarrow \left| {X_{A} \left( t \right) - X_{B} \left( t \right) \, } \right| < \tau_{{\Omega_{{X_{A} ,X_{B} }} }} $$

*Decreasing* $$ \Omega_{{X_{A} ,X_{B} }} (t) $$

$$ {\text{d}}\Omega_{{X_{A} ,X_{B} }} \left( t \right)/{\text{d}}t < 0\, \Leftrightarrow \left( {\tau_{{\Omega_{{X_{A} ,X_{B} }} }} - \left| {X_{A} \left( t \right) - X_{B} \left( t \right)} \right|} \right) < 0 \Leftrightarrow \left| {X_{A} \left( t \right) - X_{B} \left( t \right)} \right| > \tau_{{\Omega_{{X_{A} ,X_{B} }} }} $$

This shows that for cases that $\left| {X_{A} \left( t \right) - X_{B} \left( t \right) \, } \right| < \tau_{{\Omega_{{X_{A} ,X_{B} }} }}$ the connection keeps on becoming stronger until $\Omega_{{X_{A} ,X_{B} }} (t)$ approaches 1. Similarly for cases that $|X_{A} \left( t \right) - X_{B} \left( t \right)| > \tau_{{\Omega_{{X_{A} ,X_{B} }} }}$ the connection keeps on becoming weaker until $\Omega_{{X_{A} ,X_{B} }} (t)$ approaches 0. This implies that $\Omega_{{X_{A} ,X_{B} }} (t) = \, 0$ and $\Omega_{{X_{A} ,X_{B} }} (t) = { 1}$ can both become attracting, but under different circumstances concerning the values of $X_{A} \left( t \right)$ and $X_{B} \left( t \right)$. In [1], chapter 11, section 11.7 for such an adaptive network model, an example simulation is shown where indeed the connection weights all converge to 0 or 1, and during this process clusters are formed of persons with equal levels of their state; see also [32].

## Mathematical analysis for the ‘more becomes more’ principle

The criterion for stationary points applied to the adaptive network model for the ‘more becomes more’ principle is the following:

$$ {\mathbf{c}}_{A,B} \left( {\upomega_{{C_{ 1} ,B}} \left( t \right), \ldots ,\upomega_{{C_{k} , \, B}} \left( t \right)} \right) = \upomega_{A,B} \left( t \right) $$

where $C_{ 1} , \ldots ,C_{k}$, and *A* are the states connected to *B*. For a joint stationary point, this criterion applies to any state connected to *B.* Renaming *A* by $C_{k + 1}$ this can also be formulated by the following set of $k + 1$ equations for $i = { 1}, \ldots ,k + 1$:

$$ {\mathbf{c}}_{{C_{i} ,B}} \left( {\upomega_{{C_{ 1} ,B}} \left( t \right), \ldots ,\upomega_{{C_{i - 1} ,B}} \left( t \right),\upomega_{{C_{i + 1} ,B}} \left( t \right), \ldots ,\upomega_{{C_{k + 1} ,B}} \left( t \right)} \right) = \upomega_{{C_{i} ,B}} \left( t \right) $$

or written out:

$$ \begin{aligned} & {\mathbf{c}}_{{C_{{\mathbf{1}}} ,B}} (\upomega_{{C_{ 2} ,B}} (t), \ldots ,\upomega_{{C_{{k + {\mathbf{1}}}} ,B}} (t)) = \upomega_{{C_{{\mathbf{1}}} ,B}} (t) \hfill \\ & {\mathbf{c}}_{{C_{{\mathbf{2}}} ,B}} (\upomega_{{C_{{\mathbf{1}}} ,B}} (t),\upomega_{{C_{ 3} ,B}} (t), \ldots ,\upomega_{{C_{{k + {\mathbf{1}}}} ,B}} (t)) = \upomega_{{C_{2} ,B}} (t) \hfill \\ & \ldots \hfill \\ & {\mathbf{c}}_{{C_{{k + {\mathbf{1}}}} ,B}} (\upomega_{{C_{{\mathbf{1}}} ,B}} (t), \ldots ,\upomega_{Ck,B} (t)) = \upomega_{{C_{{k + {\mathbf{1}}}} ,B}} (t) \hfill \\ \end{aligned} $$

If for the combination function ${\mathbf{c}}_{\boldsymbol{{C_{i} ,B}}}$ **(..)** the scaled sum function is chosen with scaling factor the number *k*, this provides the following set of *k* + 1 linear equations for a joint stationary state for the connections to *B*:

$$ \begin{aligned} & (\upomega_{{C_{{\mathbf{2}}} ,B}} (t) + \cdots + \upomega_{{C_{{k + {\mathbf{1}}}} ,B}} (t))/k = \upomega_{{C_{{\mathbf{1}}} ,B}} (t) \hfill \\ & (\upomega_{{C_{{\mathbf{1}}} ,B}} (t) + \upomega_{{C_{ 3} ,B}} (t) + \cdots + \upomega_{{C_{{k + {\mathbf{1}}}} ,B}} (t))/k = \upomega_{{C_{2} ,B}} (t) \hfill \\ & \ldots \hfill \\ & (\upomega_{{C_{ 1} ,B}} (t) + \cdots + \upomega_{{C_{k} ,B}} (t))/k = \upomega_{{C_{{k + {\mathbf{1}}}} ,B}} (t) \hfill \\ \end{aligned} $$

By multiplying both sides by *k* this provides

$$ \begin{aligned} & (\upomega_{{C_{{\mathbf{2}}} ,B}} (t) + \cdots + \upomega_{{C_{{k + {\mathbf{1}}}} ,B}} (t)) = k\upomega_{{C_{ 1} ,B}} (t) \hfill \\ & (\upomega_{{C_{{\mathbf{1}}} ,B}} (t) + \upomega_{{C_{ 3} ,B}} (t) + \cdots + \upomega_{{C_{{k + {\mathbf{1}}}} ,B}} (t)) = k\upomega_{{C_{ 2} ,B}} (t) \hfill \\ & \ldots \hfill \\ & (\upomega_{{C_{ 1} ,B}} (t) + \cdots + \upomega_{{C_{k} ,B}} (t)) = k\upomega_{{C_{{k + {\mathbf{1}}}} ,B}} t \hfill \\ \end{aligned} $$

This set of equations can be solved easily. For each *i*, adding $\upomega_{{C_{i} ,B}} \left( t \right)$ to both sides of the *i*th equation yields

$$ \begin{aligned} & \upomega_{{C_{ 1} ,B}} \left( t \right) + \upomega_{{C_{2} ,B}} \left( t \right) + \cdots + \upomega_{{C_{{k + {\mathbf{1}}}} ,B}} \left( t \right) = k\upomega_{{C_{ 1} ,B}} \left( t \right) + \upomega_{{C_{ 1} ,B}} \left( t \right)) = (k + 1)\upomega_{{C_{ 1} ,B}} \left( t \right) \\ & \upomega_{{C_{ 1} ,B}} \left( t \right) + \upomega_{{C_{2} ,B}} \left( t \right) + \cdots + \upomega_{{C_{{k + {\mathbf{1}}}} ,B}} \left( t \right) = k\upomega_{{C_{2} ,B}} \left( t \right) + \upomega_{{C_{2} ,B}} \left( t \right) = (k + 1)\upomega_{{C_{2} ,B}} \left( t \right) \\ & \ldots \\ & \upomega_{{C_{ 1} ,B}} \left( t \right) + \upomega_{{C_{2} ,B}} \left( t \right) + \cdots + \upomega_{{C_{{k + {\mathbf{1}}}} ,B}} \left( t \right) = k\upomega_{{C_{{k + {\mathbf{1}}}} ,B}} \left( t \right) + \upomega_{{C_{{k + {\mathbf{1}}}} ,B}} \left( t \right) = \, (k + 1)\upomega_{{C_{{k + {\mathbf{1}}}} ,B}} \left( t \right). \\ \end{aligned} $$

As all left-hand sides are equal now, it follows that the right-hand sides are equal as well, so for a joint stationary point

$$ \upomega_{{C_{i} ,B}} \left( t \right) = \upomega_{{C_{j} ,B}} \left( t \right) $$

for all *i* and *j*. Therefore in a joint stationary state for all connections $\upomega_{{C_{i} ,B}}$ to *B* they have the same weight value.

By a slightly different argument a similar conclusion can be drawn when not a scaled sum combination function but a logistic combination function is chosen.

The aggregated impact on the connection weight $\upomega_{{C_{i}}, B}$ is given by

$$ \begin{aligned}&  {\mathbf{alogistic}}\left( {\upomega_{{C_{ 1} ,B}} (t), \ldots ,\upomega_{{C_{{i - {\mathbf{1}}}} ,B}} (t),\upomega_{{C_{{i + {\mathbf{1}}}} ,B}} (t), \ldots ,\upomega_{{C_{k} ,B}} \left( t \right)} \right)  \\ &\quad = \left[ {\left( {{1 \mathord{\left/ {\vphantom {1 {\left( {1 + {\mathbf{e}}^{{ - \sigma \left( {\upomega_{{C_{ 1} ,B}} + \cdots + \upomega_{{C_{i - 1} ,B}} + \upomega_{{C_{i + 1} ,B}} + \cdots + \upomega_{{C_{k} ,B}} - \tau } \right)}} } \right)}}} \right. \kern-0pt} {\left( {1 + {\mathbf{e}}^{{ - \sigma \left( {\upomega_{{C_{ 1} ,B}} + \cdots + \upomega_{{C_{i - 1} ,B}} + \upomega_{{C_{i + 1} ,B}} + \cdots + \upomega_{{C_{k} ,B}} - \tau } \right)}} } \right)}}} \right) - \left( {{1 \mathord{\left/ {\vphantom {1 {( 1+ {\mathbf{e}}^{\sigma \tau } )}}} \right. \kern-0pt} {( 1+ {\mathbf{e}}^{\sigma \tau } )}}} \right)} \right]\left( { 1+ {\mathbf{e}}^{ - \sigma \tau } } \right) \\ &\quad = \left[ {\left( {{1 \mathord{\left/ {\vphantom {1 {\left( {1 + {\mathbf{e}}^{{ - \sigma \left( {\upomega_{{C_{ 1} ,B}} + \cdots + \upomega_{{C_{i - 1} ,B}} + \upomega_{{C_{i} ,B}} + \upomega_{{C_{i + 1} ,B}} + \cdots + \upomega_{{C_{k} ,B}} - \tau - \upomega_{{C_{i} ,B}} } \right)}} } \right)}}} \right. \kern-0pt} {\left( {1 + {\mathbf{e}}^{{ - \sigma \left( {\upomega_{{C_{ 1} ,B}} + \cdots + \upomega_{{C_{i - 1} ,B}} + \upomega_{{C_{i} ,B}} + \upomega_{{C_{i + 1} ,B}} + \cdots + \upomega_{{C_{k} ,B}} - \tau - \upomega_{{C_{i} ,B}} } \right)}} } \right)}}} \right) - \left( {{1 \mathord{\left/ {\vphantom {1 {( 1+ {\mathbf{e}}^{\sigma \tau } )}}} \right. \kern-0pt} {( 1+ {\mathbf{e}}^{\sigma \tau } )}}} \right)} \right] \\ &\quad\quad \times \left( { 1+ {\mathbf{e}}^{ - \sigma \tau } } \right) \\ &\quad = \left[ {\left( {{1 \mathord{\left/ {\vphantom {1 {\left( { 1+ {\mathbf{e}}^{{ - \sigma \left( {\Omega - \upomega_{{C_{i} ,B}} } \right)}} } \right)}}} \right. \kern-0pt} {\left( { 1+ {\mathbf{e}}^{{ - \sigma \left( {\Omega - \upomega_{{C_{i} ,B}} } \right)}} } \right)}}} \right) -\upmu} \right]v \\ &\quad = \left[ {\left( {{1 \mathord{\left/ {\vphantom {1 {\left( { 1+ {\mathbf{e}}^{ - \sigma \Omega } {\mathbf{e}}^{{\sigma \upomega_{{C_{i} ,B}} }} } \right)}}} \right. \kern-0pt} {\left( { 1+ {\mathbf{e}}^{ - \sigma \Omega } {\mathbf{e}}^{{\sigma \upomega_{{C_{i} ,B}} }} } \right)}}} \right) -\upmu} \right]v \\ &\quad = \left[ {\left( {{1 \mathord{\left/ {\vphantom {1 {\left( { 1+ \kappa {\mathbf{e}}^{{\sigma \Omega_{{_{{C_{i} ,B}} }} }} } \right)}}} \right. \kern-0pt} {\left( { 1+ \kappa {\mathbf{e}}^{{\sigma \upomega_{{_{{C_{i} ,B}} }} }} } \right)}}} \right) -\upmu} \right]v \\ &\quad = {\text{f}}(\upomega_{{C_{i} ,B}} ) \\ \end{aligned} $$

with ${\text{f}}\left( V \right)$ a function defined by

$$ {\text{f}}\left( V \right) = \left[ {\frac{1}{{1 + \kappa {\mathbf{e}}^{\sigma V} }} -\upmu} \right]\nu $$

Here *κ*, *μ*, *ν* are positive constants:

$$ \begin{aligned} \kappa & = {\mathbf{e}}^{ - \sigma \Omega } {\text{ with }}\Omega = \upomega_{{C_{ 1} ,B}} + \cdots + \upomega_{{C_{k} ,B}} - \tau \\\upmu & =  1 /( 1+ {\mathbf{e}}^{\sigma \tau } ) \\ \nu & = ( 1+ {\mathbf{e}}^{ - \sigma \tau } ). \\ \end{aligned} $$

Using this function, for this case, the stationary point equations get the following uniform form:

$$ \begin{aligned} & {\text{f}}(\upomega_{{C_{ 1} ,B}} (t)) = \upomega_{{C_{ 1} ,B}} (t) \\ & \qquad \qquad  \ldots \\ & \qquad \qquad  \ldots \\ & \qquad \qquad \ldots \\ & {\text{f}}(\upomega_{{C_{k} ,B}} (t)) = \upomega_{{C_{k} ,B}} (t). \\ \end{aligned} $$

Therefore, the question becomes how many solutions the equation ${\text{f}}\left( V \right) \, = V$ has. Now ${\mathbf{e}}^{\sigma V}$ is monotonically increasing in *V*, and therefore $1/( 1+ \kappa {\mathbf{e}}^{\sigma V} ))$ and also ${\text{f}}\left( V \right)$ are monotonically decreasing: $V_{ 1} \le V_{ 2} \Rightarrow {\text{f}}\left( {V_{ 1} } \right) \ge {\text{f}}\left( {V_{ 2} } \right)$. Suppose $V_{ 1}$ and $V_{ 2}$ are two solutions of the equation ${\text{f}}\left( V \right) = V$, and assuming $V_{ 1} \le V_{ 2}$, it follows $V_{ 1} = {\text{f}}\left( {V_{ 1} } \right) \ge {\text{f}}\left( {V_{ 2} } \right) = V_{ 2} ,\;{\text{so}}\,V_{ 1} = V_{ 2}$. This implies that the equation ${\text{f}}\left( V \right) = V$ has at most one solution. From this, it follows that also for the alogistic function as combination function in a joint stationary point all $\upomega_{{C_{i} ,B}}$ values will be equal.

There is also an abstract general argument possible for a whole class of combination functions, namely, the combination functions that are (1) *symmetric* in their arguments and that are (2) *monotonic*:

1. If $U_{ 1} , \ldots ,U_{k}$ is a permutation of $V_{ 1} , \ldots ,V_{k}$, then ${\mathbf{c}}(U_{ 1} , \ldots ,U_{k} ) = {\mathbf{c}}(V_{ 1} , \ldots ,V_{k} )$
2. If it holds $U_{i} \le V_{i}$ for all *i*, then ${\mathbf{c}}(U_{ 1} , \ldots ,U_{k} ) \le {\mathbf{c}}(V_{ 1} , \ldots ,V_{k} )$

If in a fully connected network a combination function **c(..)** is used that is symmetric and monotonic and all connection weights between different states are the same (for example, assume all of them 1), and no connections occur from states to themselves, then the argument is as follows. Suppose all states have the same combination function and joint stationary points are given, so that for all *i* and *j* (assume *i* < *j*):

$$ \begin{aligned} X_{i} & = {\mathbf{c}}(X_{ 1} , \ldots ,X_{i - 1} ,X_{i + 1} , \ldots \ldots ,X_{k} ) \\ X_{j} & = {\mathbf{c}}(X_{ 1} , \ldots \ldots ,X_{j - 1} ,X_{j + 1} , \ldots ,X_{k} ) \\ \end{aligned} $$

then by symmetry

$$ \begin{aligned} X_{i} & = {\mathbf{c}}(X_{ 1} , \ldots ,X_{i - 1} ,X_{i + 1} , \ldots ,X_{j - 1} ,X_{j + 1} , \ldots ,X_{k} ,X_{j} ) \\ X_{j} & = {\mathbf{c}}(X_{ 1} , \ldots ,X_{i - 1} ,X_{i + 1} , \ldots ,X_{j - 1} ,X_{j + 1} , \ldots ,X_{k} ,X_{i} ) \\ \end{aligned} $$

Now suppose $X_{i} \le X_{j}$ then by monotonicity

$$ \begin{aligned} X_{i}  &= {\mathbf{c}}(X_{ 1} , \ldots ,X_{i - 1} ,X_{i + 1} , \ldots ,X_{j - 1} ,X_{j + 1} , \ldots ,X_{k} ,X_{j} ) \\ &  \ge  {\mathbf{c}}(X_{ 1} , \ldots ,X_{i - 1} ,X_{i + 1} , \ldots ,X_{j - 1} ,X_{j + 1} , \ldots ,X_{k} ,X_{i} ) \\ &  = X_{j} \\ \end{aligned} $$

From the above, it follows that $X_{i} = X_{j}$. The same argument applies when it is assumed $X_{i} \ge X_{j}$. Therefore in this case, in a joint stationary point all state values are equal, which was also found above by more specific methods for the special cases of a scaled sum and an advanced logistic sum combination function, which indeed both are symmetric and monotonic combination functions. Thus, we obtain the following theorem:

### **Theorem**

*When in a fully connected network with equal connection weights a combination function is used that is symmetric and monotonic, then in a joint stationary point all state values are equal.*

### ‘More becomes more’ and scale-free networks

**Fig. 7:**
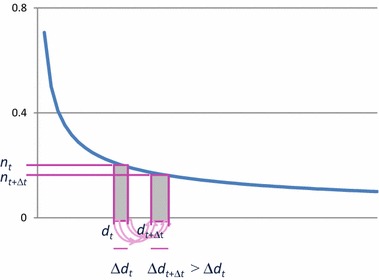
Emerging scale-free network from an adaptive network

The ‘more becomes more’ principle has also been used to provide an explanation for the empirical evidence that most real-world networks are scale-free. The idea is that the typical distribution of degrees according to a power law emerges from an evolving network when it is assumed that the network dynamics is based on some form of a ‘more become more’ principle (also called *preferential attachment*); see, for example, [30, 33–35]; see also [36, 37]. An indication of the type of argument followed is illustrated in Fig. 7. Here the distribution of nodes (vertical axis) over degrees (horizontal axis) is depicted; this distribution is assumed stable over time. A time point *t* is considered and the focus is at the nodes with some degree *d*_*t*_ at *t* (see at the horizontal axis). There is a (relative) number or density *n*_*t*_ of them (vertical axis). Moreover, the nodes with degree between *d*_*t*_ and a bit higher $d_{t} + \Delta d_{t}$ are considered, an interval of length $\Delta d_{t}$ at the horizontal axis. The (relative) number of nodes with degree within this interval is represented in Fig. 7 by the area of the (left) rectangle above that interval. This area is approximated by $n_{t} \Delta d_{t}$.

Now consider a time step from *t* to $t + \Delta t$. Due to growth of the number of connections, the nodes with degree *d*_*t*_ at time *t* will have a higher degree $d_{t + \Delta t}$ at $t + \Delta t$, and the nodes with degree $d_{t} + \Delta d_{t}$ at time *t* will have a higher degree $d_{t + \Delta t} + \Delta d_{t + \Delta t}$ at $t + \Delta t$. Due to the ‘more becomes more’ principle, from $d_{t} < d_{t} + \Delta d_{t}$ it follows that from *t* to *t* + ∆*t* the nodes with degree $d_{t} + \Delta d_{t}$ at time *t* will get more new connections than the nodes with degree *d*_*t*_ at time *t.* Therefore the increase in degree of these nodes with degree $d_{t} + \Delta d_{t}$ at time *t* will be higher:

$$ \Delta d_{t + \Delta t} > \Delta d_{t} $$

The numbers of nodes previously represented at *t* by the left rectangle are represented at $t + \Delta t$ by the right rectangle. Moreover, because they describe the same nodes, the areas indicated as shaded are the same:

$$ n_{t} \Delta d_{t} = n_{t + \Delta t} \Delta d_{t + \Delta t} $$

Given this equality, from $\Delta d_{t + \Delta t} > \Delta d_{t}$ (‘more becomes more’ principle) it follows that $n_{t + \Delta t} < n_{t}$. Therefore the distribution is monotonically decreasing. By a more complex argument it has been derived that based on some more precise assumptions on the formalisation of the ‘more becomes more’ principle, a distribution is obtained that is monotonically decreasing according to a power law; for example, see [30, 33–35] for more details.

## Discussion

The Network-Oriented Modelling approach based on adaptive temporal–causal networks, as described here (see also [1, 10]), provides a dynamic modelling approach that enables a modeller to design high-level conceptual model representations in the form of cyclic graphs (or connection matrices). These conceptual representations can be systematically transformed in an automated manner into executable numerical representations that can be used to perform simulation experiments. The modelling approach makes it easy to take into account. on the one hand. theories and findings from any domain from, for example, biological, psychological, neurological or social sciences, as such theories and findings are often formulated in terms of causal relations. This applies, among others, to mental processes based on complex brain networks, which, for example, often involve dynamics based on interrelating and adaptive cycles, but equally well it applies to the adaptive dynamics of social interactions. For a more detailed theoretical analysis on the wide applicability of the approach, see [38, 39]; for example, there it is shown that any smooth (state-determined) dynamical system can be modelled by a temporal–causal network model.

This enables to address complex adaptive phenomena within all kinds of integrated cognitive, affective and social processes. By using temporal–causal relations from those domains as a main vehicle and structure for network models, the obtained network models get a strong relation to the large body of empirically founded knowledge from the Neurosciences and Social Sciences. This makes them scientifically justifiable to an extent that is not attainable for black box models which lack such a relation.

In this paper, we have discussed in some detail how mathematical analysis can be used to find out some properties of the dynamics of a network model designed according to a Network-Oriented Modelling approach based on temporal–causal networks; see also [1], chapter 12, or [31]. An advantage is that such an analysis is done without performing simulations. This advantage makes that it can be used as an additional source of knowledge, independent of a specific implementation of the model. By comparing properties found by mathematical analysis and properties observed in simulation experiments a form of verification can be done. If a discrepancy is found, for example, in the sense that the mathematical analysis predicts a certain property but some simulation does not satisfy this property, this can be a reason to inspect the implementation of the model carefully (and/or check whether the mathematical analysis is correct). Having such an option can be fruitful during a development process of a model, as to acquire empirical data for validation of a model may be more difficult or may take a longer time.

Adaptive network models combining the homophily and the ‘more becomes more’ principle also have been studied recently, in particular in [40, 41]. The methods described in the current paper can and actually have also be applied to such integrated cases. Moreover, it has been shown in [40, 41] how the modelling approach can be related to empirical real-world data on evolving friendship networks.

Mental processes can also be modelled by temporal–causal networks in an adaptive manner. The parameters that can change can be modelled in the same way as states, following the approach in “Network-Oriented Modelling by temporal–causal networks” section. This can be applied, for example to the way in which connection strengths can change based on Hebbian learning. Hebbian learning [42], is based on the principle that strengthening of a connection between neurons over time may take place when both states are often active simultaneously (‘neurons that fire together, wire together’). The principle itself goes back to Hebb [42], but see also, e.g. [43]. For some more details on this, see [31].
